# Efeito na Qualidade de Vida de Pacientes com Insuficiência Cardíaca e Fração de Ejeção Reduzida/Preservada em Uso de Sacubitril/Valsartan

**DOI:** 10.36660/abc.20220611

**Published:** 2023-08-28

**Authors:** Yuanrui Huang, Xu Wu, Xingyu Li, Zhengzhong Liu, Yunyi Li

**Affiliations:** 1 Banan Hospital of Traditional Chinese Medicine Department of Pharmacy Chongqing China Department of Pharmacy, Banan Hospital of Traditional Chinese Medicine, Chongqing – China; 2 Banan Hospital of Traditional Chinese Medicine Department of Geriatrics Chongqing China Department of Geriatrics, Banan Hospital of Traditional Chinese Medicine, Chongqing – China; 3 Chongqing Hospital of Traditional Chinese Medicine Department of Cardiology Chongqing China Department of Cardiology, Chongqing Hospital of Traditional Chinese Medicine, Chongqing – China; 4 Banan Hospital of Traditional Chinese Medicine Department of Cardiology Chongqing China Department of Cardiology, Banan Hospital of Traditional Chinese Medicine, Chongqing – China; 5 Chongqing Hospital of Traditional Chinese Medicine Department of Pharmacy Chongqing China Department of Pharmacy, Chongqing Hospital of Traditional Chinese Medicine, Chongqing – China

**Keywords:** Insuficiência Cardíaca, Valsartana, Qualidade de Vida, Metanálise

## Abstract

**Fundamento:**

O manejo da insuficiência cardíaca (IC) tem melhorado acentuadamente, mas uma melhora clinicamente significativa na capacidade funcional e na qualidade de vida talvez seja mais importante para os pacientes do que viver mais.

**Objetivo:**

Este estudo teve como objetivo revisar a melhora na qualidade de vida com sacubitril/valsartan em pacientes com IC e fração de ejeção (FE) reduzida/preservada a partir de ensaios clínicos prospectivos.

**Métodos:**

PubMed, Embase e Cochrane Library foram pesquisados em busca de ensaios clínicos randomizados (ECRs) e estudos de coorte prospectivos publicados desde o início até julho de 2021. Um total de 6 ensaios clínicos e 16.854 pacientes com IC foram incluídos. O desfecho primário foi a alteração da linha de base na pontuação do resumo clínico do KCCQ. Os desfechos secundários foram pontuações em outros domínios do KCCQ, ocorrência de eventos adversos graves (EAs) e mortalidade geral. Valores de p < 0,05 foram considerados estatisticamente significativos.

**Resultados:**

O tratamento de sacubitril/valsartan mostrou KCCQ-CSS significativamente maior em comparação com o controle (DMP=0,975, IC 95%:0,885, 1,064, p<0,001; I^2^=94,8%, p_heterogeneidade_<0,001). Uma diminuição significativa na taxa de mortalidade foi observada no grupo sacubitril/valsartan em comparação com o grupo controle (RR=0,895, IC 95%: 0,831, 0,965, p=0,004; I^2^=43,6%, p_heterogeneidade_=0,150). No entanto, nenhuma redução significativa na ocorrência de EAs graves foi encontrada entre pacientes com IC tratados com sacubitril/valsartan em comparação com o grupo controle (RR=0,950, IC 95%: 0,879, 1,027, p<0,001; I^2^=68,1%, p_heterogeneidade_= 0,024).

**Conclusões:**

Nosso estudo demonstrou que o sacubitril/valsartan pode melhorar significativamente a QVRS em comparação com outros tratamentos de acordo com os resultados do KCCQ-CSS e alguns subdomínios do índice KCCQ durante o acompanhamento em pacientes com IC.

[Fig f1]

**Figure f1:**
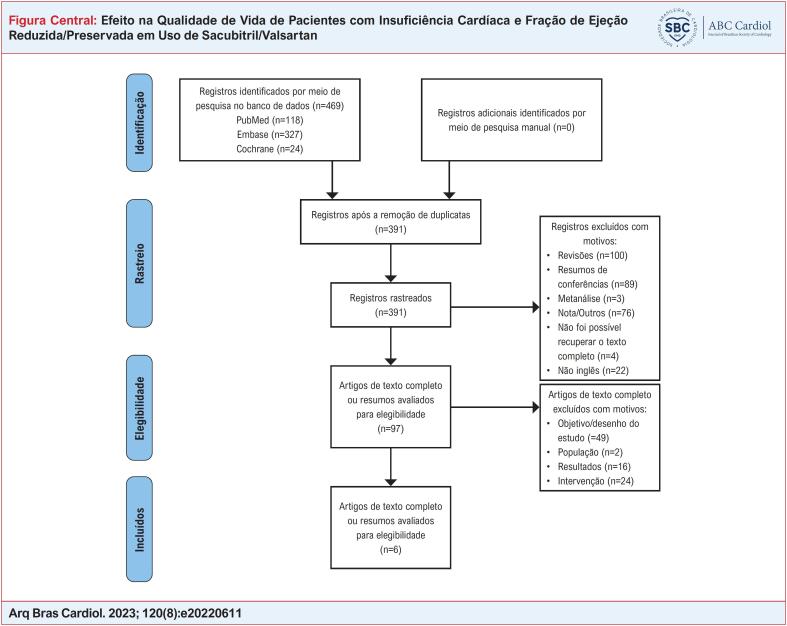
Diagrama de Fluxo do PRISMA 2009.

## Introdução

A insuficiência cardíaca (IC) é uma das principais causas de mortalidade, morbidade e hospitalizações em todo o mundo.^1^ O manejo da IC crônica melhorou acentuadamente nas últimas duas décadas com a introdução de novos procedimentos diagnósticos e terapias farmacológicas. A IC afeta negativamente a qualidade de vida relacionada à saúde (QVRS) nos domínios físico, mental e social.^2,3^ Consequentemente, a QVRS em pacientes com IC é prejudicada, mesmo quando comparada com pacientes com idade e gênero pareados com outras doenças crônicas debilitantes, como doença renal terminal em diálise.^4,5^ Muitos pacientes com IC atualmente valorizam a melhora da QVRS após o tratamento como tão importante quanto o prolongamento da vida, ou até mais.^6^ Recentemente, as diretrizes da *European Society of Cardiology* e da *American Heart Association/American College of Cardiology/Heart Failure Society of America* para o manejo da IC crônica recomendaram o uso do inibidor do receptor de angiotensina-neprilisina (ARNI) sacubitril/valsartan em pacientes com IC com fração de ejeção reduzida (ICFEr) como recomendação Classe I.^7,8^ A recomendação baseou-se nos achados robustos do maior estudo de Fase III conduzido em pacientes com ICFEr crônica, no qual o sacubitril/valsartan demonstrou ser superior ao enalapril, inibidor da enzima conversora de angiotensina (IECA), na redução da mortalidade e hospitalizações por IC, e sua melhora significativa na QVRS determinada pelo escore do *Kansas City Cardiomyopathy Questionnaire* (KCCQ) em comparação com o enalapril.^9^ Além disso, um estudo recente mostrou que o sacubitril/valsartan melhora a tolerância ao exercício.^10^ Dada a morbidade significativa associada à IC, os pesquisadores agora prestam atenção meticulosa à investigação da carga de sintomas e do efeito dos tratamentos na QVRS. Para pacientes com IC, uma melhora clinicamente significativa na capacidade funcional e na QVRS talvez seja mais importante do que viver mais, com alguns pacientes dispostos a trocar os benefícios de mortalidade ou morbidade de uma terapia por uma melhor QVRS.^11^ O KCCQ é um questionário autoaplicável e bem validado que quantifica o estado dos pacientes em vários domínios, incluindo limitações físicas, sintomas, autoeficácia, interferência/limitação social e QVRS em pacientes com IC. As pontuações no KCCQ variam de 0 a 100, com pontuações mais altas indicando menos sintomas e limitações físicas associadas à IC. O escore resumido geral do KCCQ (KCCQ-OS) captura limitação física, escore total de sintomas, QVRS e escores de limitação social; o escore resumido clínico do KCCQ (KCCQ-CSS) captura a limitação física e os escores totais de sintomas. Embora o sacubitril/valsartan tenha retardado a deterioração da QVRS no PARADIGM-HF, o momento das avaliações basais após a fase inicial e o uso de medidas subjetivas podem ter limitado a detecção de melhorias clinicamente significativas.^12^ Consequentemente, dados limitados de ensaios clínicos estão disponíveis para apoiar relatos anedóticos de melhorias clinicamente significativas na ICFEr após o início do sacubitril/valsartan.^13^ Portanto, este estudo teve como objetivo revisar a melhora na qualidade de vida com sacubitril/valsartan em pacientes com insuficiência cardíaca a partir de ensaios clínicos prospectivos.

## Métodos

### Envolvimento do paciente e do público

O conselho de ética foi consultado e declarou que nenhuma aprovação era necessária, uma vez que nenhum participante foi contatado e nenhum dado foi recuperado dos prontuários médicos.

### Busca na literatura

Esta metanálise foi conduzida de acordo com as diretrizes do *Preferred Reporting Items for Systematic Reviews and Meta-Analyses* (PRISMA).^14^ Os ensaios clínicos relevantes foram pesquisados com base no processo PICO.^15^ Uma pesquisa sistemática foi realizada no PubMed, Embase e na Cochrane Library para ECRs disponíveis publicados até julho de 2021, usando o termo MeSH ‘Heart Failure’ e ‘Quality of Life’ e palavras-chave relevantes. Para estudos que não foram publicados, mas registraram seu desenho e protocolo em ClinicalTrials.gov, nós os pesquisamos manualmente para garantir se os resultados foram publicados.

### Critério de eleição

Os critérios de elegibilidade foram: 1) população: pacientes diagnosticados com IC; 2) intervenções: tratadas com sacubitril/valsartan; 3) controle: placebo ou terapia individualizada combinada; 4) tipo de estudo: quaisquer estudos prospectivos ou ECRs publicados em revistas científicas revisadas por pares; 5) desfecho: QVRS determinada pelo escore do KCCQ; e 6) o idioma era limitado ao inglês. Informações detalhadas sobre nossas estratégias de busca podem ser encontradas nos materiais complementares.

### Extração de dados

Características do estudo (ano de publicação, país, tipo de desenho do estudo, tamanho da amostra, idade média e porcentagem masculina), parâmetros de tratamento (o nível da fração de ejeção do ventrículo esquerdo na inclusão, gravidade da IC de acordo com os critérios da *New York Heart Association*, tratamento no grupo controle, dose de tratamento), e os resultados foram extraídos por 2 autores independentemente (YR Huang e YY Li). Qualquer discrepância foi resolvida por discussão.

### Resultados

O desfecho primário foi a mudança da linha de base no KCCQ-CSS. Os desfechos secundários foram pontuações em outros domínios do KCCQ, ocorrência de eventos adversos graves (EAs) e mortalidade geral.

### Qualidade da evidência

O nível de evidência de todos os estudos incluídos foi avaliado independentemente por 2 autores (YR Huang e YY Li) usando os critérios RoB-2 ou sistema de pontuação MINORS (*Methodological Index for Non-Randomized Studies*).^16,17^ As discrepâncias na avaliação foram resolvidas por meio de discussão até que um consenso fosse alcançado.

### Análise estatística

Todas as análises foram realizadas no programa STATA SE 14.0 (StataCorp, College Station, Texas, EUA). Os resultados foram apresentados como diferenças médias ponderadas (DMP) e risco relativo (RR) sempre que apropriado. Os efeitos e respectivos intervalos de confiança de 95% (ICs) foram usados para comparar os resultados. Para estudos que não apresentaram seus resultados como média ± desvio padrão, os resultados foram estimados com base nos parâmetros relatados (mediana, IQR ou IC95%).^18^ A heterogeneidade estatística entre os estudos foi calculada usando o teste Q de Cochran e o índice I^2^. Um I^2^ >50% e um teste Q p<0,10 indicaram alta heterogeneidade, e o modelo de efeitos aleatórios foi usado; caso contrário, o modelo de efeitos fixos foi aplicado. Valores de p<0,05 foram considerados estatisticamente diferentes. A análise de sensibilidade foi realizada usando o método leave-one-out.^16^ Não avaliamos o potencial viés de publicação por gráficos de funil e teste de Egger porque o número de estudos incluídos em cada metanálise era inferior a dez, caso em que os gráficos de funil e o teste de Egger poderiam produzir resultados enganosos e não eram recomendados.

## Resultados

### Inclusão no estudo

A Figura Central apresenta o processo de inclusão no estudo. Um total de 469 estudos foram recuperados pela primeira vez e 391 estudos foram deixados após a remoção das duplicatas. Então, 294 estudos foram excluídos por causa do tipo de artigo, idioma e nenhum texto completo disponível. Dos 97 estudos restantes, após revisão dos textos completos, 49 foram excluídos por causa do objetivo/desenho do estudo, 16 pelos resultados, 2 pela população e 24 pela intervenção. Portanto, 1 estudo de coorte prospectivo e 5 ECRs foram incluídos (Tabela 1).^12,19-23^ Um total de 16.854 pacientes com IC foi incluído, com mais de 8.000 pacientes em cada grupo. O risco de viés foi baixo em todos os estudos. Um estudo^23^ que não calculou o tamanho da amostra antes do início da inscrição foi degradado de acordo com o sistema de pontuação MINORs (Material Suplementar 1).

**Tabela 1:** Características dos estudos incluídos para metanálise

Autor, ano	País	Design de estudo	FEVE na inclusão	Gravidade da IC	Controle	Tamanho da amostra		Dose		Masculino, %	Idade no grupo SV, a	Seguir
						Intervenção	Controle	Intervenção	Controle			
OUTSTEP-HF, 2020^19^	Multinacional	ECR	<40%	NYHA II/III/IV	Enalapril	302	302	50/100/200 mg por via oral duas vezes ao dia	2,5/5/10 mg por via oral duas vezes ao dia	78,7	67,16±11,04	3 meses
PARADIGM-HF, 2014^12^	Multinacional	ECR	<40%	NYHA II/III/IV	Enalapril	4187	4212	200 mg duas vezes ao dia	10mg duas vezes ao dia	78,2	63,8±11,5	8 meses
PARAGON-HF, 2019^20^	Multinacional	ECR	>45%	NYHA II/III/IV	Valsartana	2407	2389	200 mg duas vezes ao dia	160 mg duas vezes ao dia	48,3	72,7±8,3	8 meses
PARALLAX, 2021^21^	Multinacional	ECR	>40%	NYHA II/III/IV	Enalapril/Valsartana/placebo	1281	1285	50/100/200 mg por via oral duas vezes ao dia	Individualizado	49,3	N / D	6 meses
PARASAIL, 2019^22^	Canadá	Prospectivo	<40%	NYHA II/III	\	219	\	200 mg duas vezes ao dia	\	79,5	64,5±10,8	12 meses
PROVIDE-HF, 2020^23^	EUA	Prospectivo	<40%	Sem limitação	IECA /BRA	151	119	50/100/200 mg duas vezes ao dia	\	70	59 (50-67)	3 meses

FEVE: fração de ejeção do ventrículo esquerdo; IC: insuficiência cardíaca; NYHA: New York Heart Association; IECA: inibidor da enzima conversora de angiotensina; BRA: bloqueador do receptor de angiotensina; TMI: Terapia médica individualizada.

Nota: O nível de significância adaptado no PARAGON-HF para comparação do índice KCCQ foi de 0,024. Para outras comparações, valores de p <0,05 foram considerados significativos.

### Resultado primário

**Figura 1:**
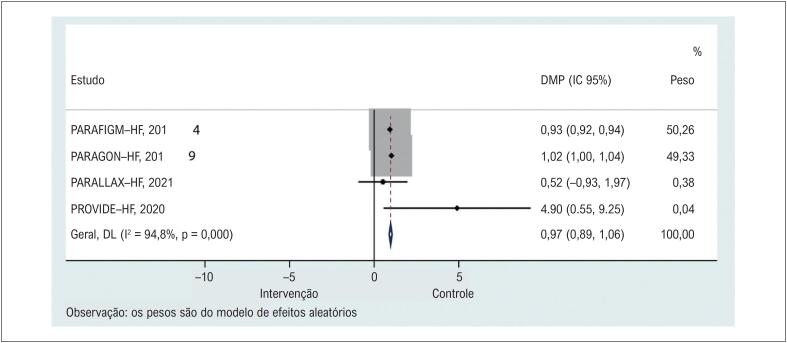
Comparação dos grupos sacubitril/valsartana e controle no KCCQ-Clinical Summary Score. DMP: diferenças médias ponderadas.

Quatro estudos^12,20,21,23^ relataram a mudança da linha de base para o acompanhamento do KCCQ-CSS nos grupos de tratamento e controle. O tratamento com sacubitril/valsartan apresentou KCCQ-CSS significativamente mais elevado do que o controle (Figura 1 e Tabela 2). As análises de sensibilidade mostraram que nenhum estudo específico contribuiu para a heterogeneidade (Material Suplementar 2).

**Tabela 2:** Resultados combinados para cada desfecho

	N	DMP (95% CI)	p (Heterogeneidade)	I-quadrado, %	p
KCCQ-CSS	4	0,975 (0,885, 1,064)	<0,001	94,8	<0,001
FEVE <40%	2	2.296 (-1.401, 5.992)	0,073	68,8	0,223
FEVE>40%	2	1,020 (0,999, 1,041)	0,5	0	<0,001
KCCQ-OS	2	2,406 (-0,826, 5,638)	0,094	64,4	0,145
Limitação física	2	0,830 (0,816, 0,844)	0,581	0	<0,001
Sintoma total	2	3,255 (-1,880, 8,389)	0,029	78,9	0,214
Auto-eficácia	2	0,790 (0,777, 0,803)	0,617	0	<0,001
Qualidade de vida	2	1.540 (1.525, 1.555)	0,152	51,2	<0,001
Limitação social	2	1.910 (1.893, 1.927)	0,501	0	<0,001
	**N**	**RR (IC 95%)**	**p (Heterogeneidade)**	**I-quadrado, %**	**p**
EA grave	4	0,950 (0,879, 1,027)	0,024	68,1	0,196
Mortalidade total	4	0,895 (0,831, 0,965)	0,15	43,6	0,004

DMP: diferenças médias ponderadas; FEVE: fração de ejeção do ventrículo esquerdo; RR: risco relativo; EA: evento adverso.

### Resultados secundários

**Figura 2:**
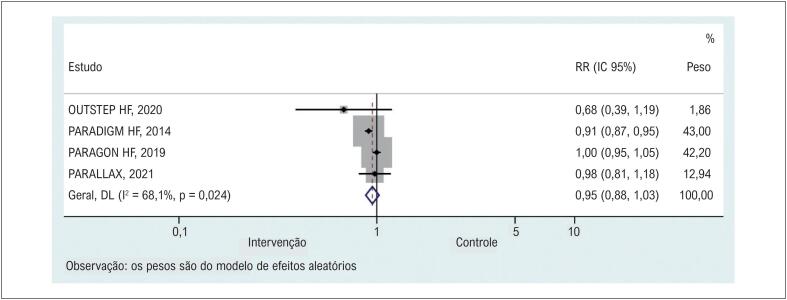
Comparação dos grupos sacubitril/valsartana e controle quanto à ocorrência de eventos adversos graves. RR: risco relativo.

Quatro estudos^12,19-21^ relataram e compararam a ocorrência de EAs graves de ambos os grupos. Os resultados combinados indicaram que o sacubitril/valsartan não reduziu significativamente a ocorrência de EAs graves em comparação com o grupo controle (Figura 2 e Tabela 2). As análises de sensibilidade mostraram que nenhum estudo específico contribuiu para a heterogeneidade (Material Suplementar 3).

**Figura 3:**
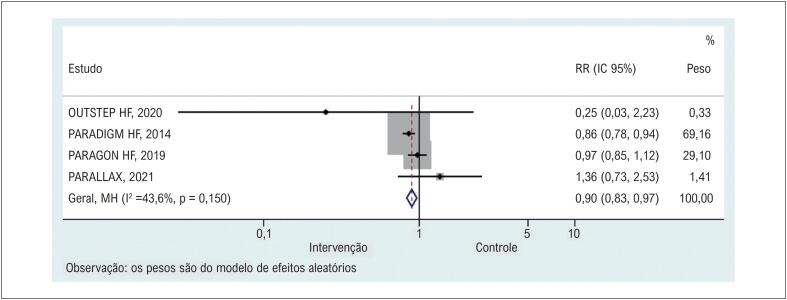
Comparação dos grupos sacubitril/valsartan e controle na mortalidade geral. RR: risco relativo.

Quatro estudos^12,19-21^ relataram a taxa de mortalidade geral. Sacubitril/valsartan diminuiu significativamente a morte por qualquer causa em comparação com o grupo controle (Figura 3 & Tabela 2). As análises de sensibilidade mostraram que nenhum estudo específico contribuiu para a heterogeneidade (Material Suplementar 4).

Os resultados de outros domínios no índice KCCQ, incluindo pontuação resumida geral, limitação física, sintoma total, autoeficácia, qualidade de vida e limitação social, foram apresentados na Tabela 2. Exceto pela pontuação resumida geral e pontuação total de sintomas (p>0,05), os resultados em outros domínios mostraram que o sacubitril/valsartan melhorou significativamente a qualidade de vida em comparação com o grupo controle. No entanto, os resultados podem não ser conclusivos, pois apenas 2 estudos^12,23^ foram incluídos nas análises.

### Análises de subgrupo de sacubitril/valsartan no KCCQ-Clinical Summary Score

**Figura 4:**
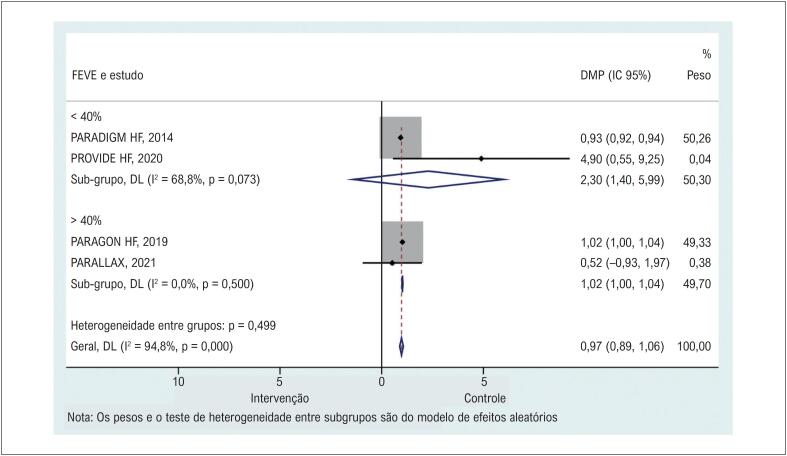
Forest plot para KCCQ-Clinical Summary Score comparando o grupo sacubitril/valsartan com o grupo controle pelo nível da fração de ejeção do ventrículo esquerdo na inclusão. DMP: diferenças médias ponderadas; FEVE: fração de ejeção do ventrículo esquerdo.

A mudança da linha de base do KCCQ-CSS não foi maior em pacientes que receberam sacubitril/valsartan em comparação com o grupo controle em pacientes com IC com FEVE <40% [12, 23], mas foi maior quando FEVE>40%^20,21^ (Figura 4 e Tabela 2).

## Discussão

A presente metanálise sugeriu que o sacubitril/valsartan melhorou significativamente a QVRS determinada pelo KCCQ-CSS para pacientes com IC e reduziu a taxa de mortalidade geral durante o seguimento. Os desfechos secundários também indicaram um efeito protetor de sacubitril/valsartan em pacientes com IC em relação à incidência de óbito. Em relação ao impacto nas atividades físicas e sociais após o tratamento com sacubitril/valsartan, resultados significativamente melhores do que o grupo controle foram observados com base nas análises de alguns subconjuntos do KCCQ.

As comparações sobre o impacto da qualidade de vida entre os tratamentos em pacientes com IC foram bastante limitadas. Estudos prospectivos anteriores com tamanho de amostra relativamente pequeno provaram que a QVRS de pacientes com IC sob tratamento de sacubitril/valsartan pode ser significativamente melhorada desde o início de acordo com o *Minnesota Living with Heart Failure Questionnaire* (MLHFQ) ou teste de caminhada de 6 minutos. No entanto, os resultados podem não ser conclusivos devido à escassez de tamanho da amostra e escalas não uniformes aplicadas em diferentes estudos.^24,25^ O KCCQ é um índice de pontuação de QVRS específico para IC, autoadministrado, validado para investigar a qualidade de vida de pacientes com IC com FE reduzida ou preservada. Nossos resultados sugeriram que o sacubitril/valsartan pode melhorar a qualidade de vida em uma pontuação de 0,975 (95% CI: 0,885 a 1,064) desde o início no KCCQ-CSS em comparação com outros tratamentos durante um acompanhamento de 3 a 8 meses. Este resultado é basicamente consistente em todos os estudos, exceto no estudo PARALLAX,^21^ que não indicou diferenças no KCCQ-CSS entre sacubitril/valsartan e monoterapia de enalapril/valsartan/placebo em 24 semanas de acompanhamento (p=0,4791). Vale a pena notar que PARALLAX é o mais recente estudo randomizado de controle com um grande tamanho de amostra (N = 2.572), e os pacientes do grupo de controle receberam tratamento designado de acordo com o tratamento anterior para comorbidades. Nesse caso, os intervalos de confiança para a alteração média da linha de base no KCCQ-CSS na semana 24 ainda eram bastante amplos e comparáveis entre os grupos, o que sugere que o impacto do sacubitril/valsartan na qualidade de vida ainda é controverso em comparação com o tratamento médico individualizado. Por outro lado, PARADIGM-HF e PARAGON-HF iniciaram o sacubitril/valsartan na dose de 200 mg duas vezes/dia, mas tanto o PARALLAX quanto o PROVIDE-HF trataram os pacientes em um nível individualizado com 50/100/200 mg duas vezes ao dia com base no tratamento anterior. Portanto, pesquisas que investiguem a dose de sacubitril/valsartan na qualidade de vida durante o acompanhamento são justificadas.

Grandes ensaios clínicos e metanálises anteriores entendem bem que sacubitril/valsartan pode reduzir significativamente a taxa de hospitalização e melhorar a capacidade funcional e a remodelação reversa cardíaca em pacientes com IC com fração de ejeção (FE) reduzida ou preservada em seguimento de curto prazo.^26-29^ O sacubitril é um inibidor da neprilisina que pode prevenir a degradação dos peptídeos natriuréticos endógenos ao aumentar as encefalinas endógenas. Além disso, a valsartana é um bloqueador do receptor de angiotensina que inibe os efeitos deletérios mediados pela angiotensina-II, incluindo vasoconstrição, hipertrofia e fibrose. Portanto, o mecanismo do efeito global do tratamento com sacubitril/valsartan é a vasodilatação, natriurese e diurese, bem como a inibição da fibrose e hipertrofia. Na análise de subgrupo do nosso estudo, os resultados indicaram uma melhora significativa no KCCQ-CSS entre os pacientes com IC e FEVE preservada (FEVE>40%) quando comparamos sacubitril/valsartan e controle, mas nenhuma diferença foi encontrada entre os pacientes com fração de ejeção reduzida (FEVE <40%). A discrepância pode vir da heterogeneidade de nossos estudos nas características demográficas, já que mais de 70% dos pacientes com IC com FE reduzida eram do sexo masculino, mas apenas 50% dos pacientes com IC com FE preservada eram do sexo masculino. Essa proporção predominantemente menor de mulheres com FE reduzida provavelmente viesou nossos resultados. Nossa premissa original é que os pacientes sob tratamento com sacubitril/valsartan teriam uma taxa de mortalidade geral predominantemente mais baixa, com ocorrência de EAs menos graves. De fato, nosso estudo confirmou o efeito protetor de sacubitril/valsartan na taxa de mortalidade geral em comparação com outros tratamentos (RR=0,90, IC 95%: 0,83 a 0,97). Entretanto, a comparação para ocorrência de EAs graves não sugeriu diferença entre os grupos (RR=0,95, IC95%: 0,88 a 1,03). Este resultado contradiz o maior ECR (PARADIGM-HF), indicando efeito protetor do sacubitril/valsartan (RR=0,91, IC95%: 0,87 a 0,95). Uma possível explicação para essa contradição é que os pacientes em PARADIGM-HF tiveram FE reduzida e receberam tratamentos em uma dose designada. Além disso, os resultados de um estudo publicado recentemente não relataram diferenças significativas entre sacubitril/valsartan e enalapril na taxa de mortalidade por todas as causas (0/69 vs. 1/70) e EAs graves (5/69 vs. 4/70) na IC pacientes com FE reduzida. Portanto, os resultados protetores de sacubitril/valsartan na morte podem ser superestimados em comparação com outros tratamentos e ainda requerem uma investigação mais aprofundada. Os resultados de um estudo publicado recentemente não relataram diferenças significativas entre sacubitril/valsartan e enalapril na taxa de mortalidade por todas as causas (0/69 vs. 1/70) e EAs graves (5/69 vs. 4/70) em pacientes com IC com FE reduzida. Portanto, os resultados protetores de sacubitril/valsartan na morte podem ser superestimados em comparação com outros tratamentos e ainda requerem uma investigação mais aprofundada.

Os resultados da presente metanálise devem ser considerados à luz das limitações do estudo. De acordo com nossas estratégias de busca, encontramos artigos avaliando a qualidade de vida por meio de várias ferramentas, como *Mini-Mental State Examination* (MMSE) para função cognitiva, *6 Minutes Walking Test* (6MWT) para função física e *Beck Depression Inventory-II* (BDI-II) e *Beck Anxiety Inventory* (BAI) para funções psicológicas (Material Complementar 5). No entanto, apenas o índice KCCQ foi usado nesta metanálise para avaliar a qualidade de vida. Em nossa defesa, apesar de várias ferramentas que podem ser aplicadas para avaliar o estado dos pacientes, o KCCQ é uma métrica de qualidade de vida relacionada à saúde bem validada em pacientes com IC e tem sido amplamente aplicada em artigos internacionais. Além disso, apenas alguns estudos utilizaram as escalas acima para avaliar a QV; é bastante difícil sintetizar seus resultados para um desfecho conclusivo. Em segundo lugar, as médias ± desvios padrão estimadas podem potencialmente enviesar os resultados, e cada estudo usou diferentes regimes e doses correspondentes em seu grupo de controle, provavelmente contribuindo para a heterogeneidade. Felizmente, a análise de sensibilidade mostrou um resultado robusto mesmo quando os estudos individuais com parâmetros estimados foram omitidos das análises. Em terceiro lugar, alguns estudos têm um tamanho de amostra bastante pequeno e um desvio padrão extremamente grande. Portanto, sua contribuição para os resultados combinados é sutil. Nesse caso, tivemos que usar o modelo de efeito aleatório para equilibrar o peso entre os grupos e moderar os efeitos predominantes de outros estudos. Quarto, apesar de sete estudos terem sido incluídos na metanálise, não mais do que quatro estudos foram analisados juntos para um determinado desfecho. Quinto, apenas artigos escritos em inglês foram incluídos, possivelmente deixando de fora resultados valiosos. Embora a diferença seja estatisticamente significativa e o tamanho da amostra em todas as análises tenha sido suficiente, a significância clínica deve ser interpretada com cautela, uma vez que os pacientes foram agrupados. Estudos adicionais podem ser necessários para determinar o impacto exato de sacubitril/valsartan na qualidade de vida de pacientes com IC.

## Conclusões

Nosso estudo demonstrou que o sacubitril/valsartan pode melhorar significativamente a QVRS em comparação com outros tratamentos de acordo com os resultados do KCCQ-CSS e alguns subdomínios do índice KCCQ durante o acompanhamento em pacientes com IC. A taxa de mortalidade foi significativamente reduzida quando comparados os pacientes tratados com sacubitril/valsartan e o regime de controle. Considerando que um ECR bem desenhado com um tamanho de amostra suficiente para investigar o efeito de sacubitril/valsartan na qualidade de vida ainda é garantido.

## *Material suplementar

Para informação adicional, por favor, clique aqui.
