# Supplementary material for: Efeito na Qualidade de Vida de Pacientes com Insuficiência Cardíaca e Fração de Ejeção Reduzida/Preservada em Uso de Sacubitril/Valsartan
Source: Arq Bras Cardiol. 2023 Aug 28;120(8):e20220611. [Article in Portuguese] doi: 10.36660/abc.20220611 (PMC10519244; doi:10.36660/abc.20220611)
Supplement: Translation

# Translation: Effect on the Quality of Life of Patients with Heart Failure and Reduced/Preserved Ejection Fraction Using Sacubitril/Valsartan

**Authors:** Yuanrui Huang, Xu Wu, Xingyu Li, Zhengzhong Liu, Yunyi Li

**Affiliations:** 1 Chongqing China Department of Pharmacy, Banan Hospital of Traditional Chinese Medicine, Chongqing - China; 2 Chongqing China Department of Geriatrics, Banan Hospital of Traditional Chinese Medicine, Chongqing - China; 3 Chongqing China Department of Cardiology, Chongqing Hospital of Traditional Chinese Medicine, Chongqing - China; 4 Chongqing China Department of Cardiology, Banan Hospital of Traditional Chinese Medicine, Chongqing - China; 5 Chongqing China Department of Pharmacy, Chongqing Hospital of Traditional Chinese Medicine, Chongqing - China

**Keywords:** Heart Failure, Valsartan, Quality of Life, Meta-Analysis

## Abstract

**Background:**

Heart failure (HF) management has markedly improved, but a clinically meaningful improvement in functional capacity and quality of life is perhaps more important for patients than living longer.

**Objective:**

This study aimed to review the improvement in quality of life with sacubitril/valsartan in patients with HF and reduced/preserved ejection fraction (EF) from prospective clinical trials.

**Methods:**

PubMed, Embase, and the Cochrane Library were searched for randomized controlled trials (RCTs) and prospective cohort studies published from inception to July 2021. A total of 6 clinical trials and 16854 patients with HF were included. The primary outcome was the change from baseline in KCCQ clinical summary score. The secondary outcomes were scores in other domains of KCCQ, the occurrence of serious adverse events (AEs), and overall mortality. P-values <0.05 were considered statistically significant.

**Results:**

Treatment of sacubitril/valsartan showed significantly higher KCCQ-CSS compared to the control (WMD=0.975, 95% CI: 0.885, 1.064, p<0.001; I^2^=94.8%, p_heterogeneity_<0.001). A significant decrease in the mortality rate was observed in the sacubitril/valsartan group compared to the control group (RR=0.895, 95%CI:0.831, 0.965, p=0.004; I^2^=43.6%, p_heterogeneity_=0.150). Nevertheless, no significant reduction in the occurrence of serious AEs was found among HF patients treated with sacubitril/valsartan compared to the control group (RR=0.950, 95%CI: 0.879, 1.027, p<0.001; I^2^=68.1%, p_heterogeneity_=0.024).

**Conclusions:**

Our study demonstrated that sacubitril/valsartan might significantly improve the HRQL compared to other treatments according to the results in KCCQ-CSS and some subdomains in the KCCQ index during the follow-up in patients with HF.

[Fig f6]

**Figure f6:** PRISMA 2009 Flow Diagram.

## Introduction

Heart failure (HF) is one of the leading causes of mortality, morbidity, and hospitalizations globally.^1^ The management of chronic HF has markedly improved over the last two decades with the introduction of novel diagnostic procedures and pharmacological therapies. HF negatively impacts health-related quality of life (HRQL) across physical, mental, and social domains.^2,3^ Consequently, HRQL in patients with HF is impaired, even when compared with age- and gender-matched patients with other debilitating chronic diseases, such as end-stage renal disease on dialysis.^4,5^ Many patients with HF currently value the improvement in HRQL after treatment as important as prolonging life, or even more.^6^ Recently, the European Society of Cardiology and the American Heart Association/American College of Cardiology/Heart Failure Society of America guidelines for the management of chronic HF recommended the use of the angiotensin receptor–neprilysin inhibitor (ARNI) sacubitril/valsartan in patients with HF with reduced ejection fraction (HFrEF) as a Class I recommendation.^7,8^ The recommendation was based on the robust findings from the largest Phase III trial conducted in patients with chronic HFrEF, in which sacubitril/valsartan was shown to be superior to the Angiotensin-Converting Enzyme Inhibitor (ACEI) enalapril in reducing mortality and HF hospitalizations, and its significant improvement in HRQL determined by Kansas City Cardiomyopathy Questionnaire (KCCQ) score compared to enalapril.^9^ In addition, a recent study showed that sacubitril/valsartan improves the tolerance to exercise.^10^ Given the significant morbidity associated with HF, researchers have now paid meticulous attention to investigating both the symptom burden and the effect of treatments on HRQL. For patients with HF, a clinically meaningful improvement in functional capacity and HRQL is perhaps more important than living longer, with some patients willing to trade the mortality or morbidity benefits of a therapy for an improved HRQL.^11^ The KCCQ is a self-administered and well-validated questionnaire that quantifies patients’ status in several domains, including physical limitations, symptoms, self-efficacy, social interference/limitation, and HRQL in patients with HF. Scores on the KCCQ range from 0 to 100, with higher scores indicating fewer symptoms and physical limitations associated with HF. The KCCQ overall summary score (KCCQ-OS) captures physical limitation, total symptom score, HRQL, and social limitation scores; the KCCQ clinical summary score (KCCQ-CSS) captures physical limitation and total symptom scores. Although sacubitril/valsartan slowed the deterioration of HRQL in PARADIGM-HF, the timing of baseline assessments after the run-in phase and the use of subjective measures may have limited the detection of clinically meaningful improvements.^12^ Consequently, limited clinical trial data are available to support anecdotal reports of clinically meaningful improvements in HFrEF after initiating sacubitril/valsartan.^13^ Therefore, this study aimed to review the improvement in quality of life with sacubitril/valsartan in patients with heart failure from prospective clinical trials.

## Methods

### Patient and public involvement

The ethical board was consulted and stated that no approval was necessary since no participants were contacted and no data was retrieved from medical charts.

### Literature search

This meta-analysis was conducted according to the Preferred Reporting Items for Systematic Reviews and Meta-Analyses (PRISMA) guidelines.^14^ The relevant clinical trials were searched based on the PICO process.^15^ A systematic search was performed from PubMed, Embase, and the Cochrane Library for available RCTs published up to July 2021, using the MeSH term ‘Heart Failure’ and ‘Quality of Life’ and relevant keywords. For studies that have not been published but registered their design and protocol in ClinicalTrials.gov, we manually searched them to ensure whether the results were posted.

### Eligibility criteria

The eligibility criteria were: 1) population: patients diagnosed with HF; 2) interventions: treated by sacubitril/valsartan; 3) control: placebo or matched individualized therapy; 4) study type: any prospective studies or RCTs published in scientific peer-reviewed journals; 5) outcome: HRQL determined by KCCQ score; and 6) language was limited to English. Detailed information on our search strategies can be found in the supplementary materials.

### Data Extraction

Study characteristics (year of publication, country, type of study design, sample size, mean age, and male percentage), treatment parameters (the level of left ventricular ejection fraction at inclusion, severity of HF according to New York Heart Association criteria, treatment in control group, dose of treatment), and outcomes were extracted by 2 authors independently (Y.R. Huang and YY Li). Any discrepancy was solved by discussion.

### Outcomes

The primary outcome was the change from baseline in KCCQ-CSS. The secondary outcomes were scores in other domains of KCCQ, the occurrence of serious adverse events (AEs), and overall mortality.

### Quality of the evidence

The level of evidence of all included studies was assessed independently by 2 authors (Y.R. Huang and YY Li) using the RoB-2 criteria or MINORS (Methodological Index for Non-Randomized Studies) scoring system.^16,17^ Discrepancies in the assessment were resolved through discussion until a consensus was reached.

### Statistical analysis

All analyses were performed using STATA SE 14.0 (StataCorp, College Station, Texas, USA). The outcomes were presented as weighted mean differences (WMD) and relative risk (RR) whenever appropriate. The effects and corresponding 95% confidence intervals (CIs) were used to compare the outcomes. For studies that did not present their results as means ± standard deviations, the results were estimated based on the reported parameters (median, IQR, or 95% CI).^18^ Statistical heterogeneity among studies was calculated using Cochran's Q test and the I^2^ index. An I^2^ >50% and a Q-test p<0.10 indicated high heterogeneity, and the random-effects model was used; otherwise, the fixed-effects model was applied. P-values <0.05 were considered statistically different. Sensitivity analysis was performed using the leave-one-out method.^16^ We did not assess the potential publication bias by funnel plots and Egger's test because the number of studies included in every meta-analysis was fewer than ten, in which case the funnel plots and Egger's test could yield misleading results and were not recommended.

## Results

### Study inclusion

The Central Figure presents the study inclusion process. A total of 469 studies were first retrieved, and 391 studies were left after removing the duplicates. Then, 294 studies were excluded because of the type of article, language, and no full text available. From the 97 studies left, after reviewing the full texts, 49 were excluded because of the study aim/design, 16 for the outcomes, 2 for the population, and 24 for the intervention. Therefore, 1 prospective cohort study and 5 RCTs were included (Table 1).^12,19-23^ A total of 16854 patients with HF were included, with over 8000 patients in each group. The risk of bias was low in all studies. One study^23^ that did not calculate the sample size before initiation of the enrollment was degraded according to the MINORs scoring system (Supplementary material 1).

**Table 1:** Characteristics of the studies included for meta-analysis

Author, year	Country	Study design	LVEF at inclusion	Severity of HF	Control	Sample size		Dose		Male, %	Age in SV group, y	Follow-up
						Intervention	Control	Intervention	Control			
OUTSTEP-HF, 2020^19^	Multinational	RCT	<40%	NYHA II/III/IV	Enalapril	302	302	50/100/200 mg orally twice daily	2.5/5/10 mg orally twice daily	78.7	67.16±11.04	3 months
PARADIGM-HF, 2014^12^	Multinational	RCT	<40%	NYHA II/III/IV	Enalapril	4187	4212	200 mg twice daily	10 mg twice daily	78.2	63.8±11.5	8 months
PARAGON-HF, 2019^20^	Multinational	RCT	>45%	NYHA II/III/IV	Valsartan	2407	2389	200 mg twice daily	160 mg twice daily	48.3	72.7±8.3	8 months
PARALLAX, 2021^21^	Multinational	RCT	>40%	NYHA II/III/IV	Enalapril/Valsartan/placebo	1281	1285	50/100/200 mg orally twice daily	Individualized	49.3	N / A	6 months
PARASAIL, 2019^22^	Canada	Prospective	<40%	NYHA II/III	\	219	\	200 mg twice daily	\	79.5	64.5±10.8	12 months
PROVIDE-HF, 2020^23^	USA	Prospective	<40%	No limitation	ACEi /ARB	151	119	50/100/200 mg twice daily	\	70	59 (50-67)	3 months

LVEF: left ventricular ejection fraction; HF: heart failure; NYHA: New York Heart Association; ACEi: angiotensin-converting enzyme inhibitor; ARB: angiotensin receptor blocker; IMT: Individualized medical therapy.

Note: The significance level adapted in PARAGON-HF for comparing the KCCQ index was 0.024. For other comparisons, p-values <0.05 were considered significant.

### Primary outcome

Four studies^12,20,21,23^ reported the change from baseline to follow-up of the KCCQ-CSS in both the treatment and control groups. Treatment of sacubitril/valsartan showed significantly higher KCCQ-CSS than the control (Figure 1 & Table 2). The sensitivity analyses showed no specific study contributed to heterogeneity (Supplementary material 2).

**Figure 1:** Comparing the sacubitril/valsartan and the control groups on KCCQ-Clinical Summary Score. WMD: weighted mean differences.

**Table 2:** Combined results for each outcome

	N	WMD (95%CI)	p (Heterogeneity)	I-square, %	p
KCCQ-CSS	4	0.975 (0.885; 1.064)	<0.001	94.8	<0.001
LVEF<40%	2	2.296 (-1.401; 5.992)	0.073	68.8	0.223
LVEF>40%	2	1.020 (0.999; 1.041)	0.5	0	<0.001
KCCQ-OS	2	2.406 (-0.826; 5.638)	0.094	64.4	0.145
Physical limitation	2	0.830 (0.816; 0.844)	0.581	0	<0.001
Total symptom	2	3.255 (-1.880; 8.389)	0.029	78.9	0.214
Self-efficacy	2	0.790 (0.777; 0.803)	0.617	0	<0.001
Quality of life	2	1.540 (1.525; 1.555)	0.152	51.2	<0.001
Social limitation	2	1.910 (1.893; 1.927)	0.501	0	<0.001
	**N**	**RR (95%CI)**	**p (Heterogeneity)**	**I-square, %**	**p**
Serious AE	4	0.950 (0.879; 1.027)	0.024	68.1	0.196
Total mortality	4	0.895 (0.831; 0.965)	0.15	43.6	0.004

WMD: weighted mean differences; LVEF: left ventricular ejection fraction; AE: adverse event; RR: relative risk.

### Secondary outcomes

Four studies^12,19-21^ reported and compared the occurrence of serious AEs from both groups. Combined results indicated sacubitril/valsartan did not significantly reduce the occurrence of serious AEs compared to the control group (Figure 2 & Table 2). The sensitivity analyses showed no specific study contributed to heterogeneity (Supplementary material 3).

**Figure 2:** Comparing the sacubitril/valsartan and the control groups on the occurrence of serious adverse events. RR: relative risk.

Four studies^12,19-21^ reported the overall mortality rate. Sacubitril/valsartan significantly decreased the death from any cause compared to the control group (Figure 3 & Table 2). The sensitivity analyses showed no specific study contributed to heterogeneity (Supplementary material 4).

**Figure 3:** Comparing the sacubitril/valsartan and control groups on the overall mortality. RR: relative risk.

Results of other domains in the KCCQ index, including the overall summary score, physical limitation, total symptom, self-efficacy, quality of life, and social limitation, were presented in Table 2. Except from the overall summary score and total symptom score (p>0.05), results in other domains showed sacubitril/valsartan significantly improved the quality of life compared to the control group. However, the results might not be conclusive since only 2 studies^12,23^ were included in the analyses.

### Subgroup analyses of sacubitril/valsartan on the KCCQ-Clinical Summary Score

The change from baseline of KCCQ-CSS was not higher in patients who received sacubitril/valsartan compared to the controls group in HF patients with LVEF<40% [12, 23], but it was higher when LVEF>40% ^20,21^ (Figure 4 and Table 2).

**Figure 4:** Forest plot for KCCQ-Clinical Summary Score comparing the sacubitril/valsartan group with the control group by the left ventricular ejection fraction level at inclusion. WMD: weighted mean differences; LVEF: left ventricular ejection fraction.

## Discussion

The present meta-analysis suggested that sacubitril/valsartan significantly improved the HRQL determined by KCCQ-CSS for patients with HF and reduced the overall mortality rate during follow-up. The secondary outcomes also indicated a protective effect of sacubitril/valsartan in patients with HF regarding the incidence of death. In terms of the impact on physical and social activities after treatment by sacubitril/valsartan, significantly better results than the control group were observed based on the analyses for some subsets of KCCQ.

Comparisons on the impact of quality of life between treatments in HF patients were rather limited. Previous prospective studies with a relatively small sample size have proved that the HRQL of HF patients under the treatment of sacubitril/valsartan can be significantly improved from baseline according to the Minnesota Living with Heart Failure Questionnaire (MLHFQ) or 6-minute walking test. However, the results might not be conclusive owing to the paucity of sample size and the nonuniform scales applied in different studies.^24,25^ KCCQ is a self-administered, HF-specific HRQL scoring index validated in investigating the quality of life for HF patients with reduced or preserved EF. Our results suggested sacubitril/valsartan can improve the quality of life by a score of 0.975 (95%CI: 0.885 to 1.064) from baseline in KCCQ-CSS compared to other treatments during a 3 to 8 months follow-up. This result is basically consistent in all studies except from the PARALLAX study,^21^ which indicated no differences in KCCQ-CSS between sacubitril/valsartan and monotherapy of enalapril/valsartan/placebo at 24 weeks of follow-up (p=0.4791). It is worth noting that PARALLAX is the latest randomized control trial with a large sample size (N=2572), and the patients in the control group received designated treatment according to their prior treatment for comorbidities. In such an instance, the confidence intervals for the mean change from baseline in KCCQ-CSS at Week 24 were still rather wide and comparable between groups, which suggested that the impact of sacubitril/valsartan on quality of life is still controversial compared to individualized medical therapy. On the other aspect, PARADIGM-HF and PARAGON-HF initiated the sacubitril/valsartan at 200 mg twice/day, but both PARALLAX and PROVIDE-HF treated the patients on an individualized level with 50/100/200 mg twice daily based on their previous treatment. Hence, research investigating the dose of sacubitril/valsartan on quality of life during follow-up is warranted.

Previous large clinical trials and meta-analyses well understand that sacubitril/valsartan can significantly reduce the hospitalization rate and improve functional capacity and cardiac reverse remodeling in HF patients with either reduced or preserved ejection fraction (EF) in short-term follow-up.^26-29^ Sacubitril is a neprilysin inhibitor that can prevent the breakdown of endogenous natriuretic peptides by increasing the endogenous enkephalins. Furthermore, valsartan is an angiotensin receptor blocker that inhibits the deleterious effects mediated by angiotensin-II, including vasoconstriction, hypertrophy, and fibrosis. Therefore, the mechanism of the overall effect of sacubitril/valsartan treatment is vasodilatation, natriuresis, and diuresis, as well as the inhibition of fibrosis and hypertrophy. In the subgroup analysis of our study, the results indicated a significant improvement in KCCQ-CSS among HF patients with preserved EF (LVEF>40%) when comparing sacubitril/valsartan and control, but no difference was found among patients with reduced EF (LVEF<40%). The discrepancy might come from our studies’ heterogeneity in demographic characteristics, as more than 70% of HF patients with reduced EF were male, yet only 50% of HF patients with preserved EF were male. This predominantly smaller proportion of women with reduced EF probably biased our results. Our original premise is that patients under the treatment of sacubitril/valsartan would have a predominantly lower overall mortality rate with less serious AEs occurring. Indeed, our study confirmed the protective effect of sacubitril/valsartan on the overall mortality rate compared to other treatments (RR=0.90, 95%CI: 0.83 to 0.97). However, the comparison for the occurrence of serious AEs suggested no difference between groups (RR=0.95, 95%CI: 0.88 to 1.03). This result contradicted the largest RCT (PARADIGM-HF), indicating a protective effect of sacubitril/valsartan (RR=0.91, 95%CI: 0.87 to 0.95). One possible explanation for this contradiction is that patients in PARADIGM-HF had a reduced EF and received treatments at a designated dose. Furthermore, results of a recently published study reported no significant differences between sacubitril/valsartan and enalapril on the all-cause mortality rate (0/69 vs. 1/70) and serious AEs (5/69 vs. 4/70) on HF patients with reduced EF. Therefore, the protective results of sacubitril/valsartan on death might be overpraised compared to other treatments and still require further investigation.

The results of the present meta-analysis must be considered in light of the study's limitations. According to our search strategies, we found articles evaluating the QoL by multiple tools such as Mini-Mental State Examination (MMSE) for cognitive function, 6 Minutes Walking Test (6MWT) for physical function, and Beck Depression Inventory-II (BDI-II) and Beck Anxiety Inventory (BAI) for psychological functions (Supplementary material 5). However, only the KCCQ index was used in this meta-analysis to assess QoL. In our defense, despite various tools that could be applied to evaluate patients’ status, the KCCQ is a well-validated health-related quality of life metric in HF patients and has been largely applied in multi-national fine articles. Moreover, only a few studies used the scales above to assess QoL; it is rather difficult to synthesize their results for a conclusive outcome. Second, the estimated means ± standard deviations might potentially bias the results, and each study used different regimens and corresponding doses in their control group, probably contributing to heterogeneity. Fortunately, the sensitivity analysis showed a robust outcome even when the individual studies with estimated parameters were omitted from the analyses. Third, some studies have a rather small sample size and extremely large standard deviation; therefore, their contribution to the combined results is subtle. In such an instance, we had to use the random-effect model to balance the weight between groups and moderate the predominant effects of other studies. Fourth, despite seven studies being included in the meta-analysis, no more than four studies were analyzed together for a given outcome. Fifth, only papers written in English were included, possibly leaving out valuable results. Although the difference is statistically significant and the sample size in all analyses was sufficient, the clinical significance should be cautiously interpreted since the patients were clustered. Additional studies might be necessary to determine the exact impact of sacubitril/valsartan on the quality of life of HF patients.

## Conclusions

Our study demonstrated that sacubitril/valsartan might significantly improve the HRQL compared to other treatments according to the results in KCCQ-CSS and some subdomains in the KCCQ index during the follow-up in patients with HF. The mortality rate was significantly reduced when comparing patients treated with sacubitril/valsartan and the control regimen. Whereas well-designed RCT with a sufficient sample size investigating the effect of sacubitril/valsartan on quality of life is still warranted.

## *Supplemental Materials

For additional information, please click here.
